# Books and Authors

**Published:** 1906-12

**Authors:** 


### BOOKS AND AUTHORS.

**American Practice of Surgery.** A Complete System of the Science and Art of Surgery by representative surgeons of the United States and Canada. Edited by **Joseph D. Bryant,** M.D., and **Albert H. Buck,** M.D., New York. Complete in eight volumes. Vol. I. Imperial octavo, pp. 818, with 294 illustrations in the text. New York: William Wood and Company. 1906. (Price, Cloth: $7.00).

The first volume of this collossal treatise on surgery makes a most favorable impression. It is a book of five parts, exclusive of the introduction which deals with the evolution of American surgery. This interesting historical résumé is prepared by Stephen Smith, who is supremely qualified for the task. He has condensed the facts most discreetly and makes a continuous story in abstract which is illustrated by wood cuts of many men eminent as surgeons in their day.

The five parts deal with surgical pathology, complications and sequelæ, general surgical diagnosis, general surgical treatment, and general surgical prognosis. The contributors are Leonard Woolsey Bacon, Joseph Colt Bloodgood, Harlow Brooks, Joseph D. Bryant, Walter J. Dodd, Harvey R. Gaylord, Preston M. Hickey, Theodore A. McGraw, James E. Moore, Albert George Nicholls, Edward Hall Nichols, Robert B. Osgood, Paul Monroe Pilcher, Stephen Smith, and Aldred Scott Warthin. We shall not undertake an exhaustive analysis of this remarkable volume, but content ourselves with a mention of some of its distinctive features.

We have already alluded to the evolution of surgery and desire to emphasise the fact that it is one of the finest bits of history in condensed form that we have seen. The second point to which we invite attention is the fact that the formation of the entire structure of written surgery, as contemplated in the scheme of this work, is laid in this volume. The topics presented pertain to the foundation of the splendid superstructure which is to be built by the seven volumes to follow. In other words, this book is devoted to the discussion of those underlying principles which constitute the basis of surgical science.

The third feature, is the monograph relating to tumors and tumor formation. This article is written by Albert G. Nicholls, of Montreal, and presents the topic in the most modern light, reflecting the newer pathology and classification of tumors, and presenting their histology in clearer perspective than has yet been done in a general treatise on surgery. It seems important that the classification herein set forth should be adopted by teachers as the simplest and easiest understood yet offered. The importance of uniformity cannot be over-accentuated, and it should be attained as nearly as may be by all instructors.

Finally, we direct attention to the section on general diagnosis which is presented by the senior editor. That accuracy in diagnosis lies at the threshold of all successful treatment, is a medical axiom that needs no elaboration here. Dr. Bryant has shown by his teachings and writings for more than twenty-five years his appreciation of diagnostic precision and has in a previous treatise laid emphasis upon this topic. It has remained for him, however, to discuss the subject here with more detail, and he has developed the refinements of method and simplicity of application with a graceful force that betrays the master. Moreover, it is a practical delineation of a topic with which every practitioner should familiarise himself, even to a degree of perfection in scientific technic not hitherto understood or appreciated.

Other important features could be taken up with profit but it is not well, in these days of hurry and worry, to overburden the reader with description. Bloodgood's article on shock, however, contains so much of value that we must not close this sketch without reference to it. Especially should his remarks on operations during shock be remembered. The mechanical construction of the book is beyond criticism and the illustrations are works of art. We cannot doubt that this treatise will recieve a degree of professional favor hitherto unaccorded any surgical work, for the reason that, judging by this volume, it will deserve it.

---

**The Medical Record Visiting List or Physicians Diary for 1907.** New York: William Wood and Company, Medical Publishers.

This excellent pocket diary enters the field early in the campaign, having come to hand during the late November days. It is one of the most compact, useful, and practical of visiting lists and is, withal, a handsome book. It may not be inappropriate to mention its contents for the information of those who have not seen former editions, which may be summed up as follows: calendar; estimation of the propable duration of pregnancy; approximate equivalents of temperature, weight, capacity, measure, etc.; maximum adult doses by the mouth, in apothecaries' and decimal measures; drops in a fluid drachm; solutions for subcutaneous injection; solutions in water for atomization and inhalation; miscellaneous facts; emergencies; surgical antiseptics; disinfection; dentition; table of signs; visiting list wth special memoranda; consultation practice; obstetric engagements; record of obstetrical practice; record of vaccination; register of deaths; nurses' addresses; addresses of patients and others; cash account.

The prices of the regular lists are: for 60 patients a week, with or without dates, handsomely selected red or black, morocco binding, $1.50; for 30 patients a week, with or without dates, same style, $1.25; also a few special 90 patient lists, $2.00. A number of novelties are offered at special prices; running even as high as $4.00 a book. Also, for stamping name in gold an extra charge of twenty-five cents is made, or for name and address fifty cents. Orders should be placed promptly to avoid delay in executing them. No physician who values his time and knows how to economise it, need be told of the great usefulness of this beautiful pocket memorandum book, diary, and visiting list.

**Progressive Medicine, Vol. VIII, No. 3, September, 1906.** A Quarterly Digest of Advances, Discoveries and Improvements in the Medical and Surgical Sciences. Edited by Hobart Amory Hare, M.D., Professor of Therapeutics and Materia Medica in the Jefferson Medical College of Philadelphia. Octavo, 298 pages, 13 illustrations. Lea Brothers & Co., Philadelphia and New York. (Per annum, in four cloth-bound volumes, $9.00; in paper binding, $6.00; carriage paid to any address).

This number opens with an instructive review of the diseases of the thorax and its viscera, including the heart, lungs, and bloodvessels, by William Ewart. He deals more particularly with the role of the lymphatic system, the early signs of pulmonary tuberculosis, the prevention of this dread malady and the treatment of tuberculosis. The vaccine method, as recommended by Wright and Urwick, introduces a perspective of possibilities, which may reasonably prove attractive to the clinician. The other reviews are, dermatology and syphilis, by William S. Gottheil; obstetrics, by Richard C. Norris; and diseases of the nervous system, by William G. Spiller. The abstract relating to the toxemia of pregnancy is one of great interest; indeed, the obstetric review is replete with valuable material. The September number maintains the reputation of this digest, heretofore established, for quality, clearness, and clinical importance.

**The Physicians' Visiting List** (Lindsay and Blakiston's) **for 1907.** Fifty-sixth year of its publication. Philadelphia: P. Blakiston's Son and Company (Successors to Lindsay and Blakiston). 1906.

This veteran visiting list is out early, thereby indicating that it keeps young, even with more than half a century of years upon its shoulders. It is, withal, one of the most complete and serviceable of its class, meeting every demand of the most exacting practice. It is published in several forms, ranging in price from $1.00 to $2.25, according to size and make-up. The dose table which it contains has been revised in accordance with the pharmacopeia of 1900, lately issued. We are always glad to greet this during its annual visitations, as it is the first one we were privileged to possess, now nearly fifty years ago. It is difficult to see how it could be improved, as now made up, and we commend it as one of the best pocket memorandum books in the market.

**Walter Reed and Yellow Fever.** By **Howard A. Kelly,** M.D., Professor of Gynecological Surgery in Johns Hopkins University. 12mo., pp. 293. Illustrated. New York: McClure, Phillips & Co. 1906.

The story of Walter Reed's life, as portrayed by his friend, the author, is a touching tribute of affection to the memory of the man who contributed as much as any American physician to the records of preventive medicine. Dr. Kelly has done a great service to the medical world in presenting in such entertaining form, a biography of this remarkable man which, incidentally, relates also to the history of yellow fever. This subject is dealt with most interestingly and is in reality a valuable contribution to its literature. Never, hereafter, in the history of the world can this infectious disease be mentioned, unless to be coupled with the name of the man who, more than any other person, contributed to its practical annihilation,—Walter Reed.

This story should be read by everyone interested in the achievements of American Medicine. It is at once a tribute to the memory of a brilliant man, and an inspiration to persistent effort. Every student of medicine in particular should read it and every intelligent citizen should familiarise hmself with "Walter Reed and Yellow Fever."

**Proceedings of the American Medico-Psychological Association,** at the sixty-first annual meeting, held at San Antonio, Texas, April 18-21, 1905. **Charles W. Pilgrim,** M.D., Secretary. Baltimore: Printed for the Association by the Friedenwald Company. 1905.

This, the oldest special medical society of national constituency, issues an annual volume of instructive interest. This one is a standard octavo of 384 pages, bound in paper, and contains many papers of scientific interest, especially to alienists. Dr. Arthur W. Hurd, of Buffalo, Superintendent of the State Hospital, contributes a paper on Korsakoff's psychosis. The president, Dr. T. W. J. Burgess, of Montreal, chose for the subject of his address, "The Insane of Canada." It appears from his statistics that there were in 1901, in the Dominion of Canada, 16,622 persons of unsound mind, a ratio of 3.125 in every thousand of population. The entire paper contains much of interest, and the whole volume is full of good material.

**International Clinics.** A quarterly of Illustrated Clinical Lectures and especially prepared articles on Treatment, Medicine, Surgery, Neurology, Pediatrics, Obstetrics, Gynecology, Orthopedics, Pathology, Dermatology, Ophthalmology, Otology, Rhinology, Laryngology, Hygiene and other topics of interest to students and practitioners. By leading members of the medical profession throughout the world. Edited by **A. O. J. Kelly,** A.M., M.D., Philadelphia. Volume II. Sixteenth series. 1905. Philadelphia and London: J. B. Lippincott Co. (Cloth, $2.00).

The "clinics" seem to grow better by age; at all events this number is one of the very best of the series. The topics are treatment, four articles; medicine, eight articles; pediatrics, one article; neurology, one article; surgery, five articles; obstetrics and gynecology, five articles; and laryngology, one article. De Lancey Rochester, of Buffalo, contributes an instructive paper on the prognosis and treatment of the chronic valvular diseases of the heart. W. A. Newman Dorland presents an elaborate paper, well illustrated, on the female perineum and its repair; and Louis Frank, of Louisville, presents two cases of ectopic pregnancy with unusual features. The entire book is most entertaining.

---

**Case Teaching in Medicine.** A series of graduated exercises in differential diagnosis, prognosis, and treatment of actual cases of disease. By **Richard C. Cabot,** A.B., M.D., (Harvard) Instructor in Medicine in the Harvard Medical School, and physician to outpatients at the Massachusetts General Hospital. Octavo, 220 pages. Boston: D. C. Heath & Co. 1906. (Price: Cloth, $1.50).

The object of this book is to aid the teacher in training his pupils to digest the material gathered from the clinic,—the author says "to think clearly, cogently, and sensibly about the data gathered by physical examination." Cabot is a teacher of experience and a logical thinker; he knows what a pupil needs and is fully capable of preparing the best for him. His book represents a plan of teaching that has proved its value, not only in his own hands but by other teachers of capability. The method is new and is worthy of further trial. Each alternate page is blank, to admit of entering notes; the book has three indexes,—one of signs and symbols, one of symptoms arranged by systems and organs, and one of diagnosis. We commend it to teacher and pupil.

---

**Transaction of the American Roentgen Ray Society.** Sixth annual meeting, held at Baltimore, September 28, 29 and 30, 1905. George C. Johnston, M.D., Secretary.

This society was organised about five years ago, and has 237 members, three of whom—namely, Drs. John T. Pitkin, Rosewell Park, and A. W. Bayliss, reside in Buffalo. The volume, a standard octavo of 224 pages and some advertisements, is well printed, contains a number of skiagraphs and other illustrations, and fairly represents the "specialty" of which it is the exponent.

**The Health-Care of the Baby.** A Handbook for Mothers and Nurses, by **Louis Fischer**, M.D., 12mo., 166 pages. New York and London: Funk & Wagnalls Co. 1906. (Price: 75 cents, net.; by mail, 82 cents).

Dr. Fischer is an experienced teacher at the New York Post-graduate medical School and Hospital and a clinician in pediatrics of successful practice. He is also the author of other books on kindred topics to this; therefore, whatever he says is entitled to full credence. The present book was prepared especially for mothers and nurses, and admirably handles the subjects of feeding in health and disease; gives directions for the management of fever, and is a guide during such diseases as measles, croup, skin diseases, etc., while it gives ample advice in cases of accidents, poisoning, and the like. The correction of bad habits, and the management of rashes, have received due consideration. It is an excellent book to put into the hands of younger mothers, whom it may save from many an anxious hour.

---

**Transactions of the Southern Surgical and Gynecological Association.** Vol. XVIII. Eighteenth Session, held at Louisville, Ky, December 12-14, 1905. Octavo, pp. 456. Edited by **William D. Haggard**, M.D., Secretary. Philadelphia: William A. Dornan. 1906.

This handsome volume attests the virility and the excellent work done by this association at its eighteenth annual meeting held at Louisville last year. The president, Dr. Lewis C. Bosher, of Richmond, as the subject of his address, discoursed upon the proper training for the practice of surgery. The theme was happily chosen and masterfully dealt with. The papers one and all are of a high order, and the discussions are able; they make the salient features of the papers stand out in strong relief. The volume is a credit to the medical profession of the south, for whose benefit the association was organised. The secretary has shown skill in editing the book.

---

**Surgical Suggestions.** Practical Brevities in Surgical Diagnosis and Treatment. By **Walter M. Brickner**, M.D., Chief of Surgical Department, Mount Sinai Hospital Dispensary, New York; Editor American Journal of Surgery; and **Eli Moschowitz**, M.D., Assistant Physician, Mount Sinai Hospital Dispensary, New York. Duodecimo; 60 pages. New York: Surgery Publishing Co. 1906. (Cloth, 50 cents).

This small volume is compiled from the surgical suggestions published in the *American Journal of Surgery* during the year 1905. It is convenient to have them at hand in this separate form, for reference when lack of time prevents the consultation of textbooks. The suggestions are epigrammatic in form and will be found pleasant reading during an idle half-hour, when concentrated study is not desirable. They are hints, too, to a further examination of topics upon which detailed information may be wanted.

**Introduction to Materia Medica and Pharmacology,** including the Elements of Medical Pharmacy, Prescription Writing, Medical Latin, Toxicology and Methods of Local Treatment. By **Oliver T. Osborne,** A.M., M.D., Professor of Materia Medica, Therapeutics and Clinical Medicine in Yale University. 12mo., 167 pp. Lea Brothers Co., Philadelphia and New York. 1906. ($1.00).

To minister to the sick,—to cure the afflicted,—is the ultimate object of the great majority of medical students. This small volume will tend to make easier the accomplishment of this general purpose. It is well arranged by a competent teacher. He says just enough on prescription writing, seeking to give hints rather than complete instruction on the subject. The suggestions relating to local or special treatments are well chosen as, indeed, are all the several topics dealt with. It can be carried in the pocket, and thus may become a companion at odd times when time must be economised.

---

**The Practical Medicine Series of Year Books.** Ten volumes. Issued under the general editorial charge of **Gustavus P. Head,** M.D., Professor of Laryngology and Rhinology in the Chicago Post-Graduate Medical School. Vol. III. The Eye, Ear, Nose, and Throat. Edited by **Casey A. Wood,** M.D., **Albert H. Andrews,** M.D., and **Gustavus P. Head,** M.D. Chicago: The Year Book Publishers. 1906. (Price, $1.50; entire series, $10.00).

It is difficult to keep pace with the ophthalmic literature of a year, but these editors have made the most of their opportunity. Ophthalmologists seem to be voluminous writers, and their branch is an important one. For these and perhaps other reasons the ophthalmic literature of even a single year is massive. Dr. Wood has devoted considerable space to the literature relating to ocular symptoms in general diseases, thus making the book of special interest to the general practitioner. The ear, nose, and throat literature has been judiciously plowed and harrowed, so that the entire volume is one of general practical value.

---

**X-Rays in General Practice. A. E. Walter,** Captain I. M. S., Superintendent of the X-Ray institute of India. Duodecimo, 174 pages. Illustrated. London: John Lane, The Bodley Head. New York: John Lane Company. 1906.

The author of this work has achieved fame in His Majesty's service in India as a medical officer. He has improved his opportunity to study the effect of *x*-rays and apply them in practice. The office of Superintendent of the *X*-Ray Institute afforded ample opportunity for the development of this special therapeutic and diagnostic measure and method.

The book is written on conservative lines and does not make extravagant claims for the *x*-ray treatment. The descriptive text is clear and the illustrations give it adequate explanation. On the whole it is one of the best manuals we have seen relating to this topic. It is one of a series of practitioners' handbooks issued by John Lane, of London and New York.

**Squibbs Materia Medica. Part I.** A complete alphabetical list of all the Squibb products, embracing the articles in the U. S. Pharmacopeia (8th Revision) and the National Formulary, together with the non-official chemicals, pharmaceuticals, and newer remedies in general use. Part II. Squibb's Medicinal Tablets, etc. A reliable and comprehensive handbook for the Physician and the Pharmacist. 1906 edition. Brooklyn: E. R. Squibb & Sons.

This is a most convenient book of reference for the busy doctor. It gives the more important data concerning the nature, source, physical and therapeutical properties, doses, antidotes, and other essentials of all the Squibb preparations and many other drugs of the pharmacopeia. Included, also, are the non-official and newer remedies. Besides, it gives a list of prices which is of importance. It will be sent to those who may not have received it, on application. This house is too well known to make further comment requisite.

---

**Transactions of the Section on Obstetrics and Diseases of Women,** of the American Medical Association at the Fifty-sixth annual session, held at Portland, Ore., July 11-14, 1905. Chicago: American Medical Association Press. 1905.

At the meeting of the section, the proceedings of which are recorded in this book, Dr. C. L. Bonifield, of Cincinnati, was chairman, and the work which he developed for the section is of great value. As the meeting was held at the far side of the continent, interest might be expected to lessen, but such seems not to have been the case. It is a good plan to publish these proceedings in separate form, so that they may become accessible to those most interested, and who have scant time to search the files of a weekly journal for the desired material.

---

**Thirty-second Annual Report of the State Board of Health of Michigan,** for the fiscal year ending June 30, 1904. **Henry B. Baker,** M.D., Secretary. Lansing: Wynkoop Hallenbeck Crawford Company, State Printers.

Michigan for many years has been one of the foremost states in its administration of public health affairs. This volume is, perhaps, less pretentious than some of its predecessors; nevertheless, it contains much of value to the sanitarian. The section dealing with consumption, with its tables and statistics, gives the impression that the subject is receiving most scentific attention by both county and state sanitary officers. The secretary has prepared his material with great care.

---

**Transactions of the Medical Association of the State of Alabama.** (The State Board of Health). Organised 1847. Meeting of 1905 held at Montgomery, April 19-22, 1905. Montgomery: Brown Printing Company. 1905.

The annual volumes of transactions of this association are well edited and well printed; besides, they always contain much of interest even for physicians living in other states. The annual oration was delivered on this occasion by Dr. Seale Harris, the subject being Fame's Temples, and in course of which he advocated the name of a physician for a place in the National Hall of Fame. The Jerome Cochrane lecture was delivered by Dr. Robert Abbe, of New York, whose subject was "The Problems of Surgery." A large and interesting group of scientific papers fills the body of the book, after which the register is printed, giving the names of the physicians in the state by counties, and a table of contents closes the volume.

---

**Blakiston's Quiz Compends.** A Compend of Obstetrics, especially adapted to the use of Medical Students and Physicians. By **Henry G. Landis,** A.M., M.D., late professor of obstetrics and diseases of women in Starling Medical College, Columbus, O. Revised and edited by **William H. Wells,** M.D., demonstrator of clinical obstetrics in Jefferson Medical College, Philadelphia. Eighth edition, illustrated. Philadelphia: P. Blakiston's Son & Co. 1906. (Price: $1.00 net).

The questions in this compend were prepared by a master and have stood the test of time. They bring out the essentials of the obstetric art and serve not only the purposes of the student, but are excellent reminders for the practitioner. Frequent revisions have served to keep the questions and answers abreast of the science as well as the art. The last three editions have been revised by Dr. Wells, who has proved equal to the important task of keeping the work fresh, and maintaining it at the highest possible mark.

---

**Annual Report of the Surgeon General of the Public Health and Marine Hospital Service of the United States,** for the fiscal year ended 1905. By **Walter Wyman,** Surgeon General. Washington: Government Printing Office. 1906.

The student of public health always finds much of interest in Surgeon-General Wyman's reports. To prevent the immigration of persons afflicted with infectious diseases, to guard against the introduction of yellow fever into our domain, to care for the hygiene and minister to the welfare of marines, to aid the quarantine authorities in divers ways,—these and many other functions devolve upon this important service. All these matters are considered in this report, as well as many others which we are unable to detail at this time,—the whole making an important and valuable contribution to our public health literature.

---

**Second Annual Report of the Henry Phipps Institute;** for the Study, Treatment and Prevention of Tuberculosis. February, 1, 1904, to February 1, 1905. **Lawrence F. Flick,** M.D., Medical Director. Published by the Henry Phipps Institute, Philadelphia: 1906.

This volume, bound in paper, contains among other valuable material an account of the work of the institute during its second year; a review of the subject of immunisation in tuberculosis; a preliminary report on the Maragliano serum treatment; and a report of some of the scientific work done by members of the staff of the institute during the year. It contains 452 pages of standard octavo text and, altogether, is one of the ablest if not the most valuable reports of institution work we have seen. Scant justice can be given to such a painstaking and useful report in the narrow space at command, but every person interested in the study of the tuberculosis problem should read or examine it with great care.

---

**Golden Rules of Surgery.** Aphorisms, Observations and Reflections on the Science and Art of Surgery. Being a Guide for Surgeons and those who would be Surgeons. By **Augustus Charles Bernays,** A.M., M.D., Heidelberg, M.R.C.S. England. 8 vo., pp. 232. St. Louis: The C. V. Mosby Medical Book Co. 1906, (Price $2.50).

This is a book of rules,—golden and otherwise,—which will be found of interest to the younger physicians. It would be somewhat confusing, however, for him to memorise the "rules" that apply to the various conditions touched upon. It is rather iconoclastic to "do away" with the term inflammation; and to substitute for it "tissue-unrest," is clumsy. The tendency nowadays is to simplify, and to "advance backward" in such crab-like fashion does not conform to the spirit of the age. The book is a curiosity that will amuse as well as instruct.

---

**Eczema.** A consideration of its course, diagnosis and treatment, with 146 prescriptions. By **Samuel Horton Brown,** M.D., Assistant Dermatologist in the Philadelphia Hospital. 12 mo. pp. 105. Philadelphia: P. Blakiston's Son & Co. 1906. (Price, $1.00).

It is a good plan to have such a monograph as this relating to so common a malady as eczema and which manifests itself in such varieties, within easy reach. Rarely have we seen so much useful information upon eczema presented in such an understandable fashion. Of course, one must know something of the clinical manifestations of the disease by personal observation, before he can apply all that the author has given in this compact little volume. The prescriptions are of the better class and can be made available by many general practitioners who almost always see these cases first. We commend the book to all such in particular.

---

**A Compend of Operative Gynecology.** Based on Lectures in the Course of Operative Gynecology on the Cadaver. Delivered by **William Seaman Bainbridge,** M.D., Adjunct Professor of Operative Gynecology on the Cadaver, New York Post-Graduate Medical School and Hospital. In Collaboration with **Harold D. Meeker,** M.D., Instructor in Operative Gynecology on the Cadaver, New York Post-Graduate Medical School and Hospital. 12mo. 76 pages. The Grafton Press, New York. (Price, $1.00).

These memoranda,—it seems to us that is the best term to apply to the groupings herein made by the author,—are of special service to younger members of the profession. They serve to refresh and to keep burnished the mental processes for both clinical application and for examination purposes. We rarely see so scientific a condensation of facts that are needed in busy practice, as have been gathered into this small volume.

---

**Blakiston's Quiz Compends.** Materia Medica, Therapeutics and Prescription Writing. By **Samuel O. L. Potter,** M.D., formerly Professor of the Principles and Practice of Medicine in the Cooper Medical College of San Francisco. Seventh edition, revised and enlarged. Philadelphia, P. Blakiston's Son & Co. 1906. (Price, $1.00).

Seven editions ought to prove the value of this compend, without further comment by journalists. Nevertheless, it is well to be reminded from time to time of the existence of such a useful presentation of the essentials of this practical topic. Students have obtained much aid from its pages and practitioners have found it a useful reminder. It can be carried in the pocket and thus be made available in an emergency of practice or for the examinations.

